# Spatiotemporal Trends and Climatic Factors of Hemorrhagic Fever with Renal Syndrome Epidemic in Shandong Province, China

**DOI:** 10.1371/journal.pntd.0000789

**Published:** 2010-08-10

**Authors:** Li-Qun Fang, Xian-Jun Wang, Song Liang, Yan-Li Li, Shao-Xia Song, Wen-Yi Zhang, Quan Qian, Ya-Pin Li, Lan Wei, Zhi-Qiang Wang, Hong Yang, Wu-Chun Cao

**Affiliations:** 1 State Key Laboratory of Pathogen and Biosecurity, Beijing Institute of Microbiology and Epidemiology, Beijing, China; 2 Shandong Center for Disease Control and Prevention, Jinan, China; 3 College of Public Health, The Ohio State University, Columbus, Ohio, United States of America; Tulane School of Public Health and Tropical Medicine, United States of America

## Abstract

**Background:**

Hemorrhagic fever with renal syndrome (HFRS) is a rodent-borne disease caused by Hantaviruses. It is endemic in all 31 provinces, autonomous regions, and metropolitan areas in mainland China where human cases account for 90% of the total global cases. Shandong Province is among the most serious endemic areas. HFRS cases in Shandong Province were first reported in Yutai County in 1968. Since then, the disease has spread across the province, and as of 2005, all 111 counties were reported to have local human infections. However, causes underlying such rapid spread and wide distribution remain less well understood.

**Methods and Findings:**

Here we report a spatiotemporal analysis of human HFRS cases in Shandong using data spanning 1973 to 2005. Seasonal incidence maps and velocity vector maps were produced to analyze the spread of HFRS over time in Shandong Province, and a panel data analysis was conducted to explore the association between HFRS incidence and climatic factors. Results show a rapid spread of HFRS from its epicenter in Rizhao, Linyi, Weifang Regions in southern Shandong to north, east, and west parts of the province. Based on seasonal shifts of epidemics, three epidemic phases were identified over the 33-year period. The first phase occurred between 1973 and 1982 during which the foci of HFRS was located in the south Shandong and the epidemic peak occurred in the fall and winter, presenting a seasonal characteristic of Hantaan virus (HTNV) transmission. The second phase between 1983 and 1985 was characterized by northward and westward spread of HFRS foci, and increases in incidence of HFRS in both fall-winter and spring seasons. The human infections in the spring reflected a characteristic pattern of Seoul virus (SEOV) transmission. The third phase between 1986 and 2005 was characterized by the northeast spread of the HFRS foci until it covered all counties, and the HFRS incidence in the fall-winter season decreased while it remained high in the spring. In addition, our findings suggest that precipitation, humidity, and temperature are major environmental variables that are associated with the seasonal variation of HFRS incidence in Shandong Province.

**Conclusions:**

The spread of HFRS in Shandong Province may have been accompanied by seasonal shifts of HTNV-dominated transmission to SEOV-dominated transmission over the past three decades. The variations in HFRS incidence were significantly associated with local precipitation, humidity, and temperature.

## Author Summary

Hemorrhagic fever with renal syndrome (HFRS), a rodent-borne disease caused by Hantaviruses, is characterized by fever, acute renal dysfunction and hemorrhagic manifestations. At present, it is endemic in all 31 provinces, autonomous regions, and metropolitan areas in mainland China where human cases account for 90% of the total global cases. Historically Shandong Province bears the largest HFRS burden in China—the cumulative number of human cases accounted for 1/3 of the national total. Here we report a spatiotemporal analysis of human HFRS cases in Shandong using reported case data spanning 1973 to 2005. Through the analysis of seasonal incidences and use of velocity maps, three phases of seasonal shifts of HFRS epidemics and the expansion pattern of HFRS endemic areas were identified over the 33-year period. In addition, precipitation, humidity, and temperature were found to be significantly associated with the seasonal variation of HFRS incidence in Shandong Province. These findings offer insights in understanding possible causes of HFRS spread and distribution and may assist in informing prevention and control strategies.

## Introduction

Hemorrhagic fever with renal syndrome (HFRS), a rodent-borne disease caused by Hantaviruses (HV), is characterized by fever, acute renal dysfunction, and hemorrhagic manifestations. HFRS, initially described clinically at the turn of the 20th century, is primarily distributed in the Asian and European continents, and worldwide approximately 150,000 to 200,000 hospitalized HFRS cases are reported each year, with the majority occurring in developing countries [1]. HFRS is widely distributed and a major public health concern in China. At present, it is endemic in all 31 provinces, autonomous regions, and metropolitan areas in mainland China where human cases account for 90% of the total global cases [2]. In China, HFRS is mainly caused by two types of Hantaviruses, i.e., Hantaan virus (HTNV) and Seoul virus (SEOV), each of which has co-evolved with a distinct rodent host [3]. HTNV, which causes a more severe form of HFRS than SEOV does, is associated with *Apodemus agrarius*, while SEOV is typically carried by *Rattus norvegicus.* Occurrence of HFRS cases is seasonal with a bimodal pattern and studies suggest that the pattern is linked to varying transmission dynamics of the two serotypes of HVs among their animal hosts - HTNV-caused HFRS cases occur year-round but tend to peak in the winter while SEOV-caused infections typically peak in the spring [4]–[8]. Historically, Shandong Province bears the largest HFRS burden in China - the cumulative human cases accounted for 1/3 of the national total [9]. This study aims to, through the use of a 33-year's (1973 to 2005) record of HFRS cases in Shandong Province, characterize the spatial and seasonal patterns of HFRS distribution and spread and explore associations between meteorological factors and such patterns of distribution and spread of the disease.

## Materials and Methods

### Study area

**Figure 1:**
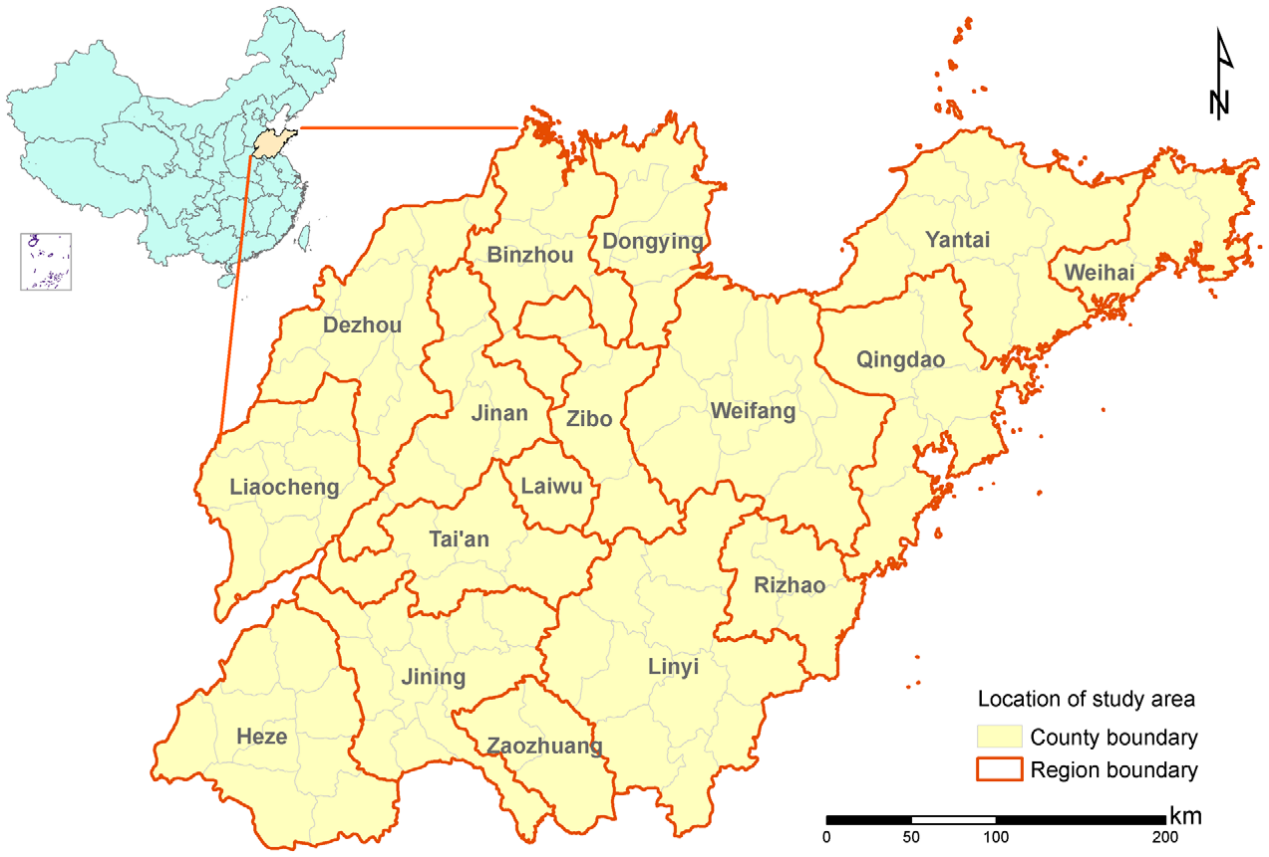
The location of study area, Shandong Province in mainland China.

The study area covers Shandong Province, a coastal province in Eastern China, located between latitude 34°25′ and 38°23′ north, and longitude 114°35′ and 112°43′ east (Figure 1). In Shandong Province, the central area is mountainous, and the eastern and the southern areas are hilly. The north and northwest parts of Shandong are composed of the alluvial plain of the Yellow River, which is part of the North China Plain. Plains and basins, mountains and hills, and rivers and lakes make up 63%, 34%, and 3% of total area of the province, respectively. The province includes 111 counties belonging to 17 regions with a total land area of 156,700 square kilometers and a population of about 90 million.

### Data collection and management

In 1950, HFRS was included on the list of Class B Notifiable Diseases in China. Since then, data reporting has followed a standard protocol determined by Chinese Center for Disease Control and Prevention and consistent throughout the country [2], [5]. Prior to 1983, a HFRS case was determined by a set of clinical criteria, as defined by a national standard. Since then, antibody-based serological tests (e.g. MacELISA, IFA) were also used and coupled with the clinical criteria [4], [7]. Clinical diagnosis criteria include: exposure history (i.e. exposure to rodents and their excreta, saliva, and urine within two months prior to the onset of illness); acute illness with at least two of the following clinical symptoms (i.e. fever, chill, hemorrhage, headache, back pain, abdominal pain, acute renal dysfunction, and hypotension); experience or partial experience of the 5 phases of disease course (i.e. fever, hypopiesis, oliguresis, hyperdiuresis, and recovery); and abnormity of blood and urine routine parameters [10]. In this study, records for HFRS cases during 1973–2005 were obtained from the Shandong Notifiable Disease Surveillance System (SNDSS) and were processed by county and by month. Disease variables (e.g. cases, deaths, and incidence of HFRS) were collected and geo-referenced on a digital map of Shandong Province using ArcGIS 9.2 (ESRI Inc., Redlands, CA, USA). Demographic data were also obtained from SNDSS. In addition, monthly meteorological data covering 700 surveillance stations in mainland China from 1973 to 2005 were collected from the China Meteorological Data Sharing Service System (http://cdc.cma.gov.cn/index.jsp). Raster format of meteorological data including monthly average temperature, relative humidity, and monthly cumulative precipitation was created using a spatial interpolation method (Kriging model). The monthly meteorological factors for each county of Shandong Province from 1973 to 2005 were then extracted in ArcGIS 9.2 (ESRI Inc., Redlands, CA, USA).

### Spatiotemporal analysis

To characterize the spatial and seasonal patterns of HFRS distribution, monthly incidences from 1973 to 2005 and for different epidemic phases in Shandong Province were plotted. Furthermore, annualized incidences and the proportion of monthly average incidence over different epidemic phases for each county were mapped in gradient colors and pie charts, respectively. To explore the diffusion trend of HFRS endemic areas, a vector velocity map [11] of HFRS spread was developed using trend surface analysis (TSA) [12]–[14]. TSA is a global smoothing method using polynomials with geographic coordinates, as defined by the central point of each county's polygon. In this study, when the first HFRS case was reported for each county, a trend surface on the month-year was created to explore the diffusion patterns and corridors of spread over time. The month-year of the first recorded case was identified for counties in the database. The x- and y-coordinates of county centroids were then derived according to an Albers conical equal area projection using the Shandong Provincial map in ArcGIS 9.2 (ESRI Inc., Redlands, CA, USA). Least square regression using quadratic polynomials of the x- and y-coordinates to predict year of first reported case was conducted using STATA 9.1 software (StataCorp LP, Texas, USA) [15]. Partial differential equations (Δyear/ΔX and Δyear/ΔY) were derived from the fitted model, generating a vector of the magnitude (i.e. slope) and direction of the diffusion trend of HFRS endemic areas for each location. The square root of the slope equates to the velocity of diffusion, as reflected by the size of the arrow on the maps.

### Meteorological factors analysis

To explore associations between the HFRS incidence and meteorological factors from 1973 to 2005, Granger causality (G-causality) tests were performed using the monthly HFRS incidence and meteorological factors (including monthly average temperature, monthly cumulative precipitation, and monthly average relative humidity) in EViews 3.1 software (Inst. of System Science, Irvine, CA, USA). To assess possible time-lag effects resulting from meteorological factors, time lags from 1 to 3 months were included in the analysis [16]. Based on the G-causality analysis at the provincial level (without reference to count), we further conducted panel data analysis at the county level. The panel data analysis was utilized to analyze the multiple cross-sectional data combining time-series and has the advantage of a large number of data points, which increased the degrees of freedom and reduced the collinearity among explanatory variables [17], [18]. In this study the panel data involved two dimensions of observations that included time series observations (monthly HFRS incidence and monthly averages of meteorological factors from 1973 to 2005) from all 111 counties in Shandong Province. The panel Poisson model with fixed effects was used to assess the impact of the three meteorological factors on HFRS incidence at varying time lags [15]. The percentage change (PC) in incidence in response to the change of a variable by a given amount (which is equal to 100*(exp (coefficient)-1)), 95% confidence intervals (CIs), and *P*-values were estimated using the maximum likelihood method. For PC estimation, a 10 mm difference was used for monthly cumulative precipitation, while 10°C and 10% differences were used for monthly average temperature and monthly average relative humidity, respectively. Univariate analysis was conducted to examine the effect of individual variables. Additionally, the time lag variables from 1 to 3 months for the three meteorological factors were tested. Multivariate analysis was then performed using variables with a *P*-value <0.1 from the univariate analysis as covariates. Correlations between covariates were quantitatively assessed and models were optimized by comparing -2 log likelihood when correlated variables were added or removed one by one. It was discovered that adding these variables with the smallest -2 log likelihood value for precipitation, humidity, and temperature, respectively, could derive a more accurate model. STATA (Version 10.0) was used in the panel data analysis (StataCorp LP, Texas, USA).

## Results

### Epidemic trend and distribution of HFRS cases

**Figure 2:**
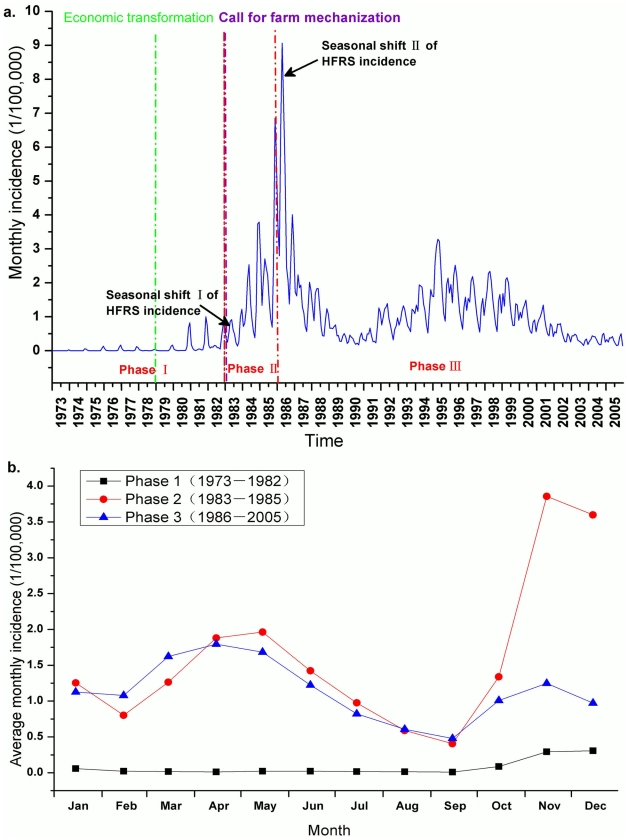
Temporal distribution patterns of HFRS incidence in Shandong Province. (a) The figure shows the monthly epidemic curve and two notable seasonal shifts of HFRS incidence from 1973 to 2005. The time of the beginning of the economic transformation and of farm mechanization was marked by green dashed and purple dashed lines, respectively. Economic transformation mainly referred to the establishment of the household responsibility contract system (e.g. holding a household fully responsible for farmland they work); the system dramatically increased farm yields and contributed enormously to the rural economy. Farm mechanization refers to the adoption of mechanized agriculture, which largely changed agricultural patterns and human behaviors. (b) The seasonal epidemic patterns for the three phases. Average monthly epidemic curves indicate the seasonal patterns of HFRS incidence and shifts of epidemic peaks of HFRS in the three phases.

The first HFRS cases in Shandong were reported in 1968 in Yutai County in the southwest region of the province, and no cases were reported again until 1973. Since then, new cases emerged in the central and southern parts of the province and endemic areas began expanding throughout Shandong Province. A total of 282,442 HFRS cases were reported in Shandong Province from 1968–2005. Because only one case was reported between 1968 and 1972, the epidemic curve was created to show the temporal distribution of HFRS from 1973–2005 in Shandong Province. The incidence curve over the 33-year period is reasonably characterized by three phases: Phase I spanning from 1973 to 1982 with a typical fall-winter peak of incidence, Phase II covering the period between 1983 and 1985 with an emerging spring peak of incidence, and Phase III spanning from 1986 to 2005 with a dominance of spring peak of incidence (Figure 2).

**Figure 3:**
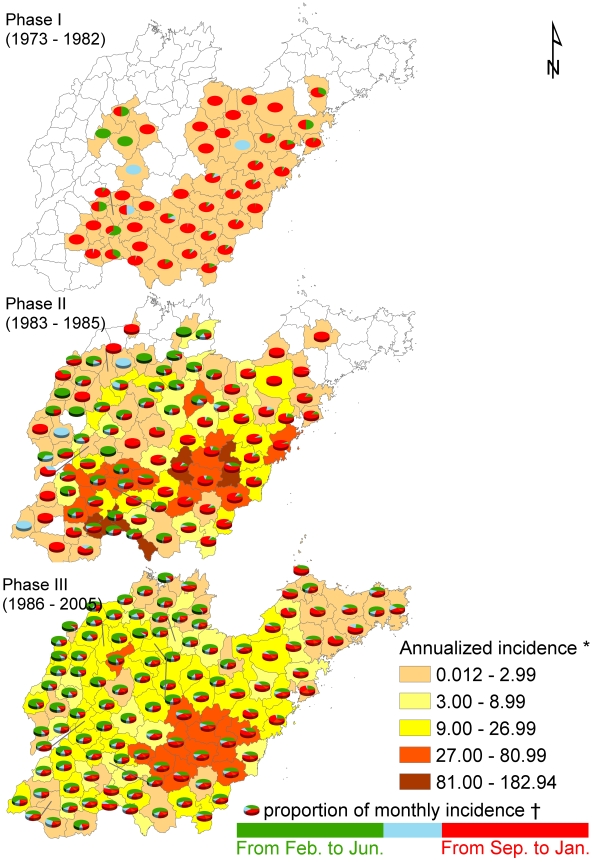
The spatial distribution of HFRS incidence and their proportion of monthly incidence in each county for three phases. The background of maps with color gradient presents the annual incidence of HFRS for each phase, and pie graphs display the proportion of monthly incidences for each county. Counties in white on the map have zero incidence. * Average annual incidence per 100,000 populations. † Proportion of average monthly incidence in these pie graphs, where green color indicates the proportion of average monthly incidence from February to June (in spring and early summer), light blue is the proportion of average monthly incidence from July to August (in summer), and the red represents the proportion of average monthly incidence from September to January (in autumn and winter).

Figure 2a shows the monthly epidemic curve from 1973 to 2005 in Shandong while Figure 2b shows seasonal shifts of incidence distributions during the three phases. Clearly a bimodal pattern of incidences is seen during phase II and III where a rapid increase of HFRS incidence was shown in both the spring and the fall-winter season during phase II. During phase III the incidence in the spring season continued at approximately the same level, but then declined quickly in the fall-winter season. Figure 3 shows annualized incidences and the proportion of monthly average incidence over three epidemic phases for each county. During phase I, the main endemic areas of HFRS are located in south-central Shandong Province with a single epidemic peak in the fall-winter season mapped by the red color of the pies. In phase II, newly established endemic areas emerged in northwest and southwest Shandong Province, an epidemic peak in the spring season is mapped by the green color in the pies (Figure 3). In phase III, we found that the epidemic peak in the spring season remained predominant in almost all counties in Shandong Province (Figure 3).

### The spatial trend surface of expansion of HFRS endemic areas

**Figure 4:**
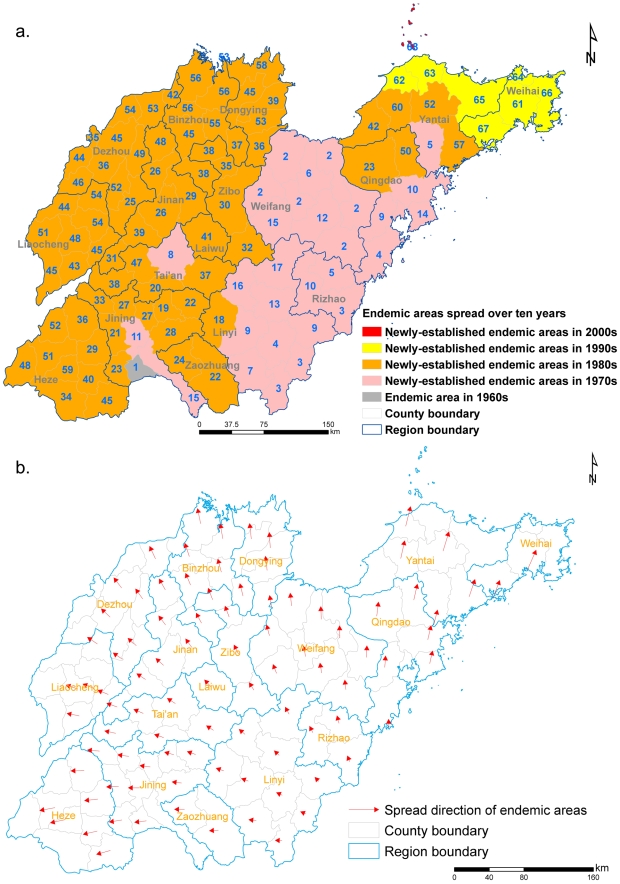
Spatial trends of HFRS expansion in endemic areas of Shandong Province from 1968 to 2005. (a) The spatial dynamics of endemic areas of HFRS per decade presented by color gradients from 1968 to 2005 in Shandong Province, the numbers represent orders ranked by the month of first case reported in these counties. (b) The vector diagram of the spatial spread of endemic areas. Arrows and their lengths present the expansion direction of HFRS endemic areas and the average annual speed of diffusion of each location since the year where HFRS cases were reported, respectively.

Figure 4 summarizes the spatial trend of expansion of HFRS endemic areas in Shandong Province. Figure 4a shows that expansion of HFRS endemic areas over the past decades since the 1960s as shown in different colors and the number in each county indicates the order ranked by the month-year of the first recorded case in the county. The velocity and direction of diffusion for each coordinate location were mapped to show the movement and instantaneous rate of HFRS diffusion in Shandong Province over the study period. Trend surface analysis with high-order polynomials is sensitive to data anomalies at the edge of the study area [11]. Less data are available at the study area boundaries; velocity vector size and direction are therefore less reliable and may not be accurate at the edge of the study area. For these reasons, 18 velocity vectors were removed from the vector diffusion map. Velocity of movement was lowest early in the epidemic (Figure 4b), when HFRS spread from its primary focus in southern Shandong Province. The epidemic diffused outward with a higher velocity of movement from the south-center Shandong Province in 1980s. The disease moved distinctly north, east, and west into all counties. These results are consistent with detection of cases in 2005 in the northernmost region of Shandong Province.

### Meteorological factors contributing to HFRS incidence

Pairwise G-causality analysis showed the association between HFRS incidence and meteorological factors with different time lags at the provincial level (Table 1). The results indicated that precipitation and humidity are G-causalities of HFRS incidence, as significant one-way association was seen between meteorological factors and the HFRS incidence but not vice versa. The association between the average temperature and HFRS incidence is not clear from this analysis, as a mixed picture was seen (i.e. two-way association). In the panel data analysis, univariate analysis showed the effects of monthly cumulative precipitation, monthly average relative humidity, monthly average temperature and their time-lag variables from 1 to 3 months separately, and indicated that all variables, except for monthly average relative humidity with the 3-month lag, appeared to be significant factors (Table 2). Multivariate panel data analysis demonstrated that the three variables, monthly cumulative precipitation with 1-month lag, monthly average relative humidity with 1-month lag and monthly average temperature with 2-month lag, were significantly associated with HFRS incidence (Table 2).

**Table 1:** Pairwise Granger causality tests between HFRS incidence and meteorological factors with different time lags in Shandong Province.

Granger causality	1-month lag		2-month lag		3-month lag	
	F-Statistic	P value	F-Statistic	P value	F-Statistic	P value
Precipitation→HFRS incidence	8.20	0.004	7.88	<0.001	10.19	<0.001
HFRS incidence→Precipitation	0.53	0.465	3.50	0.031	1.69	0.169
Humidity → HFRS incidence	5.77	0.017	10.05	<0.001	10.79	<0.001
HFRS incidence→ humidity	0.95	0.331	2.73	0.067	0.72	0.542
Average temperature→ HFRS incidence	4.47	0.035	1.51	0.222	3.72	0.012
HFRS incidence →Average temperature	2.90	0.090	6.40	0.002	7.71	<0.001

**Table 2:** Association between HFRS incidence and meteorological factors by panel data analysis.

Meteorological factors(Unit)	Univariate analysis		Multivariate analysis	
	Crude PC(95% CI)	P-value	Adjusted PC(95% CI)	P-value
Monthly cumulative precipitation (10 mm)				
no time lag	−3.31(−3.37∼−3.25)	<0.001		
1-month lag	−4.45(−4.52∼−4.38)	<0.001	−3.01(−3.10∼−2.92)	<0.001
2-month lag	−3.13(−3.19∼−3.07)	<0.001		
3-month lag	−0.73(−0.79∼−0.68)	<0.001		
Monthly average relative humidity (10%)				
no time lag	−20.16(−20.47∼−19.85)	<0.001		
1-month lag	−22.92(−23.22∼−22.62)	<0.001	−11.60(−12.12∼−11.07)	<0.001
2-month lag	−15.50(−15.83∼−15.18)	<0.001		
3-month lag	−0.29(−0.67∼0.09)	0.135		
Monthly average temperature (10°C)				
no time lag	−10.96(−11.30∼−10.63)	<0.001		
1-month lag	−16.36(−16.68∼−16.04)	<0.001		
2-month lag	−18.47(−18.78∼−18.16)	<0.001	−0.99(−1.51∼−0.47)	<0.001
3-month lag	−16.36(−16.68∼−16.05)	<0.001		

## Discussion

This study, utilizing a longitudinal dataset spanning 33 years, characterized the spatiotemporal distribution and three phases of HFRS epidemics in Shandong Province. Over the past three decades, HFRS endemic areas have spread from their initial centers - Rizhao, Linyi, and Qingdao regions - towards the northern, western, and eastern parts of the province. Notably, shifts of seasonal peaks of HFRS, as characterized by the three epidemic phases, are suggested to be associated with shifts of causal agents of HFRS - HTNV and SEOV, each with different epidemiological characteristics. For example, the HFRS epidemic shift from phase I (1973 – 1982) to phase II (1983–1985) suggest that SEOV may have emerged as dominant causal agent for the spring peak of HFRS incidence. Early studies reported that the transmission of Hantaviruses through *A. agrarius* mice peaks in the winter, while *R. norvegicus* rat-associated infections mainly occurred in the spring [4]–[7]. Given the well-documented evidence and the three-phase pattern observed in the present study, we infer that HFRS in phase I (1973–1982) and phase II (1983–1985) was primarily caused by HTNV in southeastern Shandong, and SEOV was responsible for the HFRS epidemics after phase II in most areas of Shandong Province. The seasonal and spatial distributions of HFRS cases by these two agents are consistent with the distributions of relevant rodent species - *A. agrarius* was found predominantly on the eastern coast, and *R. norvegicus* was distributed in almost all areas of Shandong Province according to surveillance studies of HFRS in China since the 1980s [6],[7],[19] Although cross-protective immunity to hantaviruses exists in recovered humans, the type of HFRS endemic areas largely depends on predominant reservoirs and their populations. In mainland China, antigen-positive *A. agrarius*, *Apodemus peninsulae*, and *R. norvegicus* rats remain predominant in rural areas, forest areas, and urban areas, respectively [8]. Noticeably, the newly-established HFRS endemic areas in mainland China since the 1990s, also including those in Shandong Province, are mostly associated with SEOV (e.g. peridomestic rodents-associated) with a characteristic spring peak of human infections [4],[20],[21]. The results suggest that prioritizing control efforts on peridomestic rodents in residential areas in the spring and on sylvatic rodents in the late autumn and early winter might provide an effective method to target the specific Hantaviruses that causes HFRS.

Our meteorological factor analysis shows monthly cumulative precipitations with 1-month lag, monthly average relative humidity with 1-month lag, and monthly average temperature with 2-month lag are significantly associated with the seasonal variation of HFRS incidence in Shandong Province. As we know, rodent populations, the reservoirs of HFRS, respond rapidly to conducive weather conditions [22]. The relationship between rodent population dynamics and meteorological factors is complex, varying by different rodent species and climate regions [23]. These complicated relationships may have different influences on disease transmission. For example, heavy precipitation followed by increased grass seed production was associated with higher deer mouse densities that caused an outbreak of hantavirus pulmonary syndrome in the Four Corners region of the USA (the New Mexico area) [24]–[27]. However, excessive rainfall could have a negative impact on rodents by destroying their habitats in Eastern China [28], [29]. In addition, frequent rain may decrease the likelihood of rodent-to-rodent contact, rodent-to-human contact, and virus transmission due to decreased rodent activity and reduced human exposure [28]. However, underlying mechanisms for the negative correlations between HFRS incidence, temperature, and relative humidity are not yet clear. As a whole, HFRS incidence in the most recent past two decades was highest in the frigid-temperate zone, mostly in northeastern China, followed by the warm-temperate zone, with lower incidence seen in southeastern China, where there are higher temperatures, higher humidity, and greater precipitation [2], [8]. This is consistent with the results of our analyses of meteorological factors. Thus, we assume meteorological factors may affect rodent dynamics and activity as well as infectivity of Hantavirus. The results support further research related to rodent host ecology. The current study suggests that climate may be used as a predictor of the intensity of HFRS transmission in a larger geographical area, however, future research is needed to better understand the underlying mechanistic effects, in particular those related to rodent ecology.

The transmission of Hantaviruses to humans is also related to other factors such as human activities, farming patterns, and rodent abatement strategies [30]. HVs are primarily transmitted from rodent hosts to humans by aerosols generated from contaminated wastes (e.g. urine and feces) of rodents, and to a lesser extent, possibly by contaminated food or rodent bites [31], [32]. We speculate that socio-environmental changes in the past three decades may have impacted human-rodent interactions. From the 1970s to the 2000s, rural areas in Shandong, like many areas in the rest of country, experienced economic transformation and farm mechanization (Figure 2a). Phase I described in this study occurred during the beginning of this transformation, possibly reflecting greater exposure to rodents in the field during the extensive farming activities. During this time, crop storage in households was relatively rare due to the commune system. HFRS cases during this time were probably largely due to sylvatically acquired HTNV. During phase II, from 1983 to 1985, higher farm output and crop yield resulted in contacts between human and rodents both in fields and in residences. In addition, relatively less precipitation during this phase might have increased the likelihood of rodent-to-human contact and virus transmission [27]. Therefore, HFRS incidence in this phase reflected more or less equally high both in the fall-winter and in the spring. For phase III of the HFRS incidence after 1986, the shift of manual to mechanized farming and increased storage of crop/food in households resulted in a greater frequency of human-rodent interactions in residences, thus causing a high spring and low winter pattern of incidence. In addition, the annual incidence of HFRS declined from 26.0 to 3.6 per 100,000 people during the 1995–2005 period, which may be related to improved housing conditions, better environmental sanitation, a transformation of farm mechanization in rural area, and control measures, in addition to the influence from climate variation in which the negative effects on incidence from monthly cumulative precipitations with 1-month lag, monthly average relative humidity with 1-month lag, and monthly average temperature with 2-month lag, which were assessed during this phase within this area (results not shown). Furthermore, our results indicating an association between HFRS incidence and meteorological factors with time lags indicate that these meteorological factors may also influence the populations and infection rates of Hantan viruses in rodents via the gestation periods and sexual maturation of rodents (which is typically 2–3 months) [33].

Despite insights gained from the present study, the limitations of our study should also be acknowledged. Firstly, due to a lack of time series data on the Hantaviruses of HFRS cases, population densities of rodents, and other influencing factors, it is difficult to further uncover the probable causes of the shifts of seasonal patterns of HFRS incidence from 1973 to 2005. Secondly, although the accuracy of clinically diagnosed HFRS cases is high (greater than 80%), atypical cases of HFRS and mistaken diagnoses of HFRS patients infected by Seoul virus existed [5], the increase of incidence beginning from Phase II also could be affected by the change from purely clinical to laboratory based surveillance in that years, when the availability of laboratory testing could encouraged more clinicians to look for the disease, and possible biases in disease reporting could influence the analysis of our results. In addition, the data are from a passive surveillance system. As a result, cases might be underreported, which then might influence the precision of our analyses. Also, several issues remain unaddressed: how changes in land use, housing conditions, and lifestyles might influence the spread of HFRS in the province and how these factors impact transmission dynamics associated with each serotype? Such knowledge is particularly relevant in the context of the continued spread of HFRS endemic areas throughout Shandong Province, as well as the variation in the seasonal distribution of HFRS incidence. Understanding these issues may further assist in informing prevention and control strategies.

## Supporting Information

Alternative Language Abstract S1 Translation of the Abstract into Chinese by Li-Qun Fang
(0.04 MB DOC)
