# Supplementary material for: Spatiotemporal Trends and Climatic Factors of Hemorrhagic Fever with Renal Syndrome Epidemic in Shandong Province, China
Source: PLoS Negl Trop Dis. 2010 Aug 10;4(8):e789. doi: 10.1371/journal.pntd.0000789 (PMC2919379; doi:10.1371/journal.pntd.0000789)
Supplement: Alternative Language Abstract S1 — Translation of the Abstract into Chinese by Li-Qun Fang (0.04 MB DOC) [file pntd.0000789.s001.doc]

**山东省肾综合征出血热流行的时空趋势及气象因素研究（摘要）**

**方立群^1,‡^ 王显军^2,‡^ 梁松^3,‡^ 李艳丽^1^ 宋韶霞^2^ 张文义^1^**

**钱全^1^ 李亚品^1^ 卫兰^1^ 王志强^2^ 杨红^1^ 曹务春[[1]](#footnote-2),***

**背景：**肾综合征出血热是由汉坦病毒引起的一种鼠传疾病，目前在中国内地31个省、自治区和直辖市均有病例报告，病例数占全球报告病例数的90%。山东省作为中国肾综合征出血热最严重的疫区之一，从鱼台县1968年报告首例病例以来，至2005年已扩散至全省，省内111个县均有肾综合征出血热病例的报告，导致其快速扩散及广泛分布的原因尚不明确。

**方法与结果：**本研究应用山东省1973-2005年肾综合征出血热发病数据开展其时空分布分析，建立肾综合征出血热季节性分布地图和疫源地空间扩散地图，并应用面板数据分析方法研究肾综合征出血热发病率与气象因素的关系。结果显示山东省肾综合征出血热疫区由最初山东省南部的临沂、日照和潍坊地区向北、向东和向西扩散，并发现山东省肾综合征出血热在上述33年间存在三个流行阶段：第一阶段（1973～1982年），肾综合征出血热疫区主要分布于山东省南部地区，表现为秋冬季单峰型疫区的特点，显示出汉滩型病毒感染的季节分布特征；第二阶段（1983～1985年），疫区向北、向西扩展，秋冬季与春季发病率同时上升，春季流行高峰显示出汉城型病毒感染的季节分布特征；第三阶段（1986～2005年），疫区向山东省东北方向扩展并蔓延至全省，秋冬季发病率迅速下降，春季发病仍保持较高水平。另外，本研究还发现降雨、湿度和温度是山东省肾综合征出血热发病率存在季节性差异的主要环境影响因素。

**结论：**过去30年间山东省肾综合征出血热疫源地发生变迁的同时，其流行季节也发生了变化，这种变化提示山东省由最初的汉滩型病毒主导的感染向汉城型病毒导致感染的转变。山东省肾综合征出血热发病率的变化与当地的降雨、湿度和气温显著相关。

1. 作者单位：1 病原微生物生物安全国家重点实验室，军事医学科学院微生物流行病研究所；2 山东省疾病预防控制中心；3 俄亥俄州大学公共卫生学院；‡ 共同为第一作者；* 通讯作者：曹务春，北京市丰台区东大街20号，北京微生物流行病研究所病原微生物生物安全国家重点实验室；邮箱地址：[caowc@nic.bmi.ac.cn](mailto:caowc@nic.bmi.ac.cn) [↑](#footnote-ref-2)
